# 92. Adult onset chronic recurrent multifocal osteomyelitis (CRMO): experience from Bahrain

**DOI:** 10.1093/rap/rky032

**Published:** 2018-09-20

**Authors:** Fatima Haji, Zahra Ali

**Affiliations:** Rheumatology, Salmanya Medical Hospital, Manama, BAHRAIN

**Introduction:** Chronic nonbacterial osteomyelitis (also called chronic recurrent multifocal osteomyelitis [CRMO]) is a rare autoimmune inflammatory disorder. Generally, CRMO is a pediatric disease, with a mean age at onset of 10 years old and it is seen more frequently in girls. Several diseases have been reported to accompany CRMO, including palmoplantar pustulosis, psoriasis, Crohn's disease, acne, and Sweet syndrome. In addition, implying that SAPHO syndrome was given its acronymic name based upon the presence of synovitis, acne, pustulosis, hyperostosis, and osteitis in the patients described in early reports. It is a rare inflammatory disorder of bone, joints, and skin, which was first described as a syndrome in 1987 adult-onset CRMO should be included in the definition of SAPHO syndrome. Indeed, there are only a few reports of adult patients with SAPHO syndrome in which the phenotype was CRMO without any skin manifestations. We herein report three cases of adult-onset CRMO presenting with inflammatory back pain, peripheral arthritis and osteitis with bony swelling of the clavicles. Two of them lacked presence of acne or pustular skin rash and one with pustular rash over hands and found to be associated with IBD.

**Case description:** Case 1: A 38 year old male, originally from Yamen, not known to have any medical illness, presented with one year history of inflammatory lower back pain more on the left side, anterior chest pain, and pain and swelling of both clavicles. His had early morning stiffness for about 15-30 minutes. There was no history of acne, psoriasis, uveitis, fever, STDs, or diarrhea. He had negative family history to similar conditions. Physical examination revealed both clavicular head and costovertebral joints tenderness and swelling, left sacroiliac joint tenderness and tender swelling over the shaft of the right tibia. He was seen earlier by another rheumatologist and was diagnosed as ankylosing spondylitis, for which salazopyrin 1 gm twice daily started for three months with no improvement, then was referred to our hospital for follow up. At the time of presentation in our hospital his laboratory tests showed normal complete blood count, normal liver function tests, C-reactive protein was 35.5 mg/dl, ESR of 32 mm/hour, His HLA-B27 was negative. Bone scan demonstrated increased uptake within both clavicles, the manubrio-sternal junction, several costochondral junctions, the left sacroiliac joint, right tibial tuberosity and subtalar joints. MRI sacroiliac joint showed left sacroilitis. CT chest abdomen and pelvis revealed erosive changes along the medial ends of both clavicles and sternoclavicular joints. Complete workup to role out infection and malignancy was negative. He was started on methotrexate 15mg per week with no response after three months. Patient then was started on adalimumab. After two months follow up he showed a very good response with about 80-90% improvement in his symptoms, C-reactive protein 0.9 mg/dland ESR 2 mm/hour.

Case 2: A 47 year old Bahraini female, known to have diabetes type 2 on oral hypoglycemic which was controlled. Presented with three years history of lower inflammatory back pain more on the left side, neck pain, and intermittent pain of small joints of the hands pain. Also had painful swelling of both clavicles. There was significant early morning stiffness lasting few hours. She was not able to perform her duties as a housewife. There was no history of acne, psoriasis, or any other skin rash, nor history of uveitis, fever, STDs, or diarrhea. She had negative family history to similar conditions. Physical examination revealed both clavicular head tenderness and swelling, and left sacroiliac joint tenderness. Otherwise examination of the peripheral joints was unremarkable. She was seen earlier by another rheumatologist and was diagnosed as ankylosing spondylitis, for which salazopyrin 1 gm twice daily and methotrexate 20 mg per week started but failed to control her symptoms, for which she was referred to our hospital for follow up. At the time of presentation in our hospital her laboratory tests showed normal complete blood count, normal liver function tests, C-reactive protein was 1.5 mg/dl, ESR of 31 mm/hour. Her HLA-B27 was negative. MRI clavicle showed bone marrow edema over sternal and clavicular parts of right sternoclavicular joint with osseous irregularity and small osteophyte with new bone formation, and .MRI sacroiliac joint showed unilateral left sacroilitis (image 2). She was started on etanercept. After about three months she showed a good response with about 50-60% improvement in her symptoms, C-reactive protein 0.5 mg/dland ESR 20 mm/hour.

Case 3: A 19 year old Bahraini male, not known previously to have any medical illness presented with three months history of large joints pain and swelling (both wrists, knees, and ankles), with bilateral jaw pain and inflammatory lower back pain. He has 1-2 hrs early morning stiffness. He also complained of abdominal pain for the same period, associated with non-bloody diarrhea 3-5 times per day, vomiting, un-intentional weight loss (5-6 kg) and low grade episodic fever. Also gave history of sudden onset acne over his face and arms for which he seeks dermatologist opinion and have been treated with oral retinoid without any improvement. He had negative history of psoriasis, uveitis, other bony pain or swellings, nor family history for psoriasis or inflammatory arthritis. On physical examination he had active synovitis of both wrists and both knees with effusion, positive Patrick's test for both sacroiliac joints, cystic acne over his cheeks and pustular rash over the dorsum of both hands, shoulders, elbows, and the back. His laboratory tests were haemoglobin 12 g/dl, WBC 7.95 x 109/L, platelets 410 x 109/L, normal liver and renal functions, ESR 96 mm/hr, C-reactive protein 44.7 mg/L, negative HLA B-27, negative serology for acute EBV and CMV infections, synovial fluid was inflammatory with total WBCs of 5000, with 72% PNLs and 28% Lymphocytes. He underwent Gastrocolonoscopy which showed grossly pancolitis with superficial erosions and pangastritis. Histopathology of gastric and colonic biopsy showed chronic focally active inflammation with erosions and cryptitis, mild gastritis and dudenititis. MRI of sacroiliac joint showed bilateral symmetrical inflammatory sacroilitis. CT chest, abdomen and pelvis was negative for malignancy, CT chest cut demonstrated both clavicular erosive changes. His workup for infection was also negative.

His final diagnosis was SAPHO associated with IBD. He was started by the gastroenerologist on pulse methylprednislone followed by oral prednisolone tapered over six weeks, with mesalazine and then adalimumab was started. After two months follow up he showed dramatic response with resolution of all of his symptoms including the skin rash. Repeated ESR was 4 mm/hr and C-reactive protein was 0.63 mg/L.

**Discussion:** CRMO is mainly seen in children, with only a few reports of patients with adult-onset CRMO. SAPHO syndrome covers a broad spectrum of findings and it is diagnosed when a patient has as few as one of the four following nonspecific features: joint lesions accompanying severe acne; joint lesions accompanying palmoplantar pustulosis; osteohypertrophy of the extremities, spine, or sternocostoclavicular joints; and CRMO. Since CRMO is one of the criteria for SAPHO syndrome, regardless of the presence or absence of skin lesions, Two of our patients with CRMO could also have been diagnosed with SAPHO syndrome. However, skin manifestations are a major feature of SAPHO syndrome and CRMO without skin lesions complicates its diagnosis. In two of our patients initial presentation was with inflammatory back pain and unilateral sacroilitis with no evidence of septic arthritis. One patient had bilateral sacroilitis and evidence of IBD but had pusutlar lesions suggestive of SAPHO. All patients had no family history of inflammatory arthritis and their HLA B27 was negative in all.

**Key Learning Points**: CRMO should be considered in adults with inflammatory arthritis in the absence of acne or pustular skin rash. Bone Biopsies can be avoided if criteria of CRMO applied. Prompt diagnosis can avoid complications if started treatment earlier.

**Disclosure: F. Haji:** None. **Z. Ali:** None.

